# The Protective Role of Interleukin-33 in Myocardial Ischemia and Reperfusion Is Associated with Decreased HMGB1 Expression and Up-Regulation of the P38 MAPK Signaling Pathway

**DOI:** 10.1371/journal.pone.0143064

**Published:** 2015-11-16

**Authors:** Ma Ruisong, Hu Xiaorong, Hu Gangying, Yi Chunfeng, Zhang Changjiang, Li Xuefei, Li Yuanhong, Jiang Hong

**Affiliations:** 1 Department of Cardiology, Renmin Hospital of Wuhan University; Cardiovascular Research Institute of Wuhan University, Wuhan, China; 2 Department of Cardiology, Central Hospital of Enshi Tujia and Miao Autonomous Prefecture, Enshi City, Hubei, China; Indiana University School of Medicine, UNITED STATES

## Abstract

Interleukin-33 (IL-33) plays a protective role in myocardial ischemia and reperfusion (I/R) injury, but the underlying mechanism was not fully elucidated. The present study was designed to investigate whether IL-33 protects against myocardial I/R injury by regulating both P38 mitogen-activated-protein kinase (P38 MAPK), which is involved in one of the downstream signaling pathways of IL-33, and high mobility group box protein 1 (HMGB1), a late pro-inflammatory cytokine. Myocardial I/R injury increased the level of IL-33 and its induced receptor (sST) in myocardial tissue. Compared with the I/R group, the IL-33 group had significantly lower cardiac injury (lower serum creatine kinase (CK), lactate dehydrogenase (LDH), and cTnI levels and myocardial infarct size), a suppressed inflammatory response in myocardial tissue (lower expression of HMGB1, IL-6, TNF-α and INF-γ) and less myocardial apoptosis (much higher Bcl-2/Bax ratio and lower cleaved caspase-3 expression). Moreover, IL-33 activated the P38 MAPK signaling pathway (up-regulating P-P38 expression) in myocardial tissue, and SB230580 partially attenuated the anti-inflammatory and anti-apoptosis effects of IL-33. These findings indicated that IL-33 protects against myocardial I/R injury by inhibiting inflammatory responses and myocardial apoptosis, which may be associated with the HMGB1 and P38 MAPK signaling pathways.

## Introduction

Myocardial reperfusion therapies, which are the most effective treatments for acute myocardial infarction, include percutaneous coronary intervention (PCI), thrombolytic therapy and coronary artery bypass grafts (CABGs). Although reperfusion therapy is critical for the survival of ischemic myocardial tissue, reducing the infarct size and improving myocardial function, the subsequent ischemia and reperfusion (I/R) injury may attenuate the therapeutic benefit. Myocardial I/R injury occurs in the early state of reperfusion, gradually changes from reversible damage to irreversible damage, and is a leading cause of death from myocardial infarction. Thus, the prevention and treatment of myocardial I/R injury has become one of the most important therapies of acute myocardial infarction.

The inflammation induced by I/R is one of the key pathophysiological processes in myocardial I/R injury [1–3]. In the inflammatory process, various cytokines are released, such as HMGB1, tumor necrosis factor α (TNF-α), and interleukin 6 (IL-6), and inflammatory cells, such as neutrophils, are activated. In addition, vascular endothelial cell injury and adhesion molecules are involved in the inflammatory response. All of these processes cause myocardial cell damage.

IL-33 was identified as a new IL-1 family member in 2005 [4]. IL-33, a non-chromosomal nuclear protein that may regulate gene transcription and cell proliferation [5], could be passively released by necrotic or apoptotic cells; when combined with its specific receptor (ST2), IL-33 could function as an inflammatory cytokine. A growing body of evidence has suggested that IL-33 plays a protective role in many cardiovascular diseases, including myocardial I/R injury, by suppressing inflammatory cytokine expression and inflammatory responses [6–8]. Further, HMGB1 is a critical inflammatory cytokine in myocardial I/R injury. The release of HMGB1 can worsen myocardial I/R injury, and inhibiting its release can reduce myocardial I/R injury. In addition, previous studies have indicated that IL-33 can regulate the HMGB1 expression level [9–12]. Thus, we hypothesize that IL-33 protects against myocardial I/R injury by inhibiting inflammatory responses, including HMGB1 expression. The present study tests this hypothesis and the possible mechanism in a rat myocardial I/R model.

## Materials and Methods

### Animal Preparation and Experimental Design

All experimental protocols in this study conformed to the Guidelines for the Care and Use of Laboratory Animals published by the US National Institutes of Health (NIH Publication, revised 1996) and were approved by the Renmin Hospital of Wuhan University. Male Sprague–Dawley rats (200–250 g) were assigned to the following six treatment groups using a random number table:

Group 1 sham-operated control + sterile saline (SO) (n = 10): rats were subjected to surgical manipulation without the induction of myocardial ischemia. After being anesthetized, the rats were treated with sterile saline (0.5ml per rat, i.v., tail vein)
Group 2 ischemia and reperfusion + sterile saline (I/R) (n = 10): rats were subjected to left anterior descending coronary artery (LAD) occlusion for 30 min followed by reperfusion for 4 h. After being anesthetized, the rats were treated with sterile saline (0.5ml per rat, i.v., tail vein) 30 minutes before LAD occlusion.
Group 3 IL-33+I/R (IL-33+I/R) (n = 6): rats were subjected to LAD occlusion for 30 min followed by reperfusion for 4 h. After being anesthetized, the rats were treated with IL-33 (10 μg per rat, i.v., tail vein, PEPROTECH, USA) [8] 30 minutes before LAD occlusion. IL-33 was dissolved in sterile saline.
Group 4 IL-33+I/R+anti-ST2 (n = 6): rats were subjected to LAD occlusion for 30 min followed by reperfusion for 4 h. After being anesthetized, the rats were treated with IL-33 (10 μg per rat, i.v., tail vein) 30 min before LAD occlusion. Anti-ST2 (0.2 mg per rat, Bios, Peking) was injected via the opposite tail vein.
Group 5 IL-33+I/R+SB203580 (IL-33+I/R+SB) (n = 6): rats were subjected to LAD occlusion for 30 min followed by reperfusion for 4 h. After being anesthetized, the rats were treated with IL-33 (10 μg per rat, i.v., tail vein) 30 min before LAD occlusion. SB203580 (an inhibitor of P38 MAPK, 1 mg/kg, Sigma-Aldrich, USA) was injected via the opposite tail vein. IL-33 and SB203580 were both dissolved in sterile saline.
Group 6 I/R + anti-ST2 (n = 6): Rats were subjected to LAD occlusion for 30 min followed by reperfusion for 4 h. After being anesthetized, anti-ST2 (0.2 ml per rat, i.v., tail vein, Bios, Peking) was injected 30 min before LAD occlusion.

The rats were anesthetized with 2.5% sodium pentobarbital (45 mg/kg, ip).

The I/R model in rats was used as previously described [13].

After 4 h of reperfusion, rats were anesthetized with a half dose (22.5 mg/kg, i.p.) of 2.5% sodium pentobarbital. Next, 2 ml of blood was collected from the jugular vein, and then the heart was excised quickly. The excess parts were removed; thus, only the infarct area (white) and risk area (5 mm around the infarct area) remained and were immediately frozen at -80°C for subsequent assays. The personnel who performed these assays were blinded to the treatment allocation.

### Myocardial Injury

The serum levels of lactate dehydrogenase (LDH) and creatine kinase (CK) were determined to assess myocardial injury. Blood samples were collected and centrifuged. Standard techniques using commercialized assay kits according to the manufacturer’s instructions (Nanjing Jiancheng Bioengineering Institute, China) were used for the analyses. The values are expressed in international units (U) per liter.

### Infarct size

Infarct size was determined using a previously described double-staining technique [13]. Briefly, after reperfusion, the LAD was occluded again, and 2 ml of 1.0% Evans blue dye was injected via the femoral vein. The heart was frozen (-80°C, 15 min) and sliced into five slices. The slices was incubated in 1.0% 2,3,5-triphenyltetrazolium chloride (TTC, Sigma-Aldrich) at 37°C for 15 min. The infarct area (white) and the area at risk (red) from each section were photographed using an image analyzer (Image-Pro Plus 6.0, Media Cybernetics, Silver Spring, MD). Infarct size was expressed as the following percentage: infarct area/ (risk+ infarct) area.

### ELISA

The expression levels of TNF-α, INF-γ and IL-6 in myocardial tissue and cTnI in the serum were assessed by a commercial ELISA kit (TNF-α, INF-γ and IL-6: Nanjing Jiancheng Bioengineering Institute, China; cTnI: Elabscience Biotechnology Co., Ltd, Wuhan, China), according to the manufacturer’s instructions.

### TUNEL assay

Cardiac myocyte apoptosis was assessed by terminal deoxynucleotidyl transferase dUTP nick end labeling (TUNEL). Myocardial trusses from the I/R, IL-33±I/R and IL-33±SB230580±I/R groups were fixed, embedded, sectioned, deparaffinized and rehydrated. Apoptosis was detected using a commercial TUNEL kit (Roche Applied Science, Indianapolis, USA), and the operation was exactly in accordance with manufacturer’s instructions. Sections were stained using a DAB staining kit (yellow) (Wuhan Boster Bioengineering Institute, China). Apoptotic nuclei were dyed brown, and normal nuclei were blue. Five fields at ×200 magnification were randomly selected from each group, and the apoptotic index (AI) was calculated. AI was defined as (number of brown nuclei per field/total number of nuclei per field) × 100%.

### Western blot

Myocardial tissue and cardiac myocyte protein expression were assessed with western blot assays as described previously [14]. Briefly, tissue or cell lysates were resolved on SDS–polyacrylamide gels (PAGE) and transferred to polyvinylidene fluoride membranes. After being blocked with 5% non-fat milk, the membranes were probed with primary antibodies, including anti-IL-33 and anti-sST (diluted 1:800, Santa), anti-p-P38 and anti-P38 (diluted 1:800, Bioworld), Bcl2 (diluted 1:500, Santa), anti-Bax (diluted 1:800, Bioworld), anti-HMGB1 (diluted 1:2000, Baster), anti-cleaved caspase-3 (diluted 1:100, Wuhan Mitaka Biotechnology Co, Ltd), and β-actin (Wuhan Boster Bioengineering Co, Ltd). After incubation with the appropriate secondary antibodies, the specific bands were visualized with an ECL detection system according to the manufacturer’s instructions.

### Quantitative PCR

The mRNA expression levels of IL-33, sST, HMGB1, Bcl-2 and Bax were analyzed by quantitative PCR as previously described [15]. The total RNA was extracted from cells, and 0.2 μg of RNA was analyzed. The sequences of the primers used are listed in Table 1.

**Table 1:** Real-time PCR primers. F: forward primers, R: reverse primers.

gene	Sequence	manufacturer
**b-240**	F: CACGATGGAGGGGCCGGACTCATC	GenScript (Nanjing) Co., Ltd.
	R: TAAAGACCTCTATGCCAACACAGT	
**IL-33**	F: AGGTAGCAAGCATGAAGGGA	GenScript (Nanjing) Co., Ltd.
	R: GTCGTTGTATGTGCTCAGGG	
**sST**	F: ATGCTGTCCTGCCGTCTCCA	GenScript (Nanjing) Co., Ltd.
	R: GGCTCCAGGGCATCGTTCTC	
**HMGB1**	F: TGGTGATGTTGCGAAGAAAC	GenScript (Nanjing) Co., Ltd.
	R: TTCATCCTCCTCGTCGTCTT	
**Bcl-2**	F: CTGGCATCTTCTCCTTCCAG	GenScript (Nanjing) Co., Ltd.
	R: CGGTAGCGACGAGAGAAGTC	
**Bax**	F: CAGGCGAATTGGCGATGAAC	GenScript (Nanjing) Co., Ltd.
	R: CCCAGTTGAAGTTGCCGTCT	

### Statistical analysis

SPSS 17.0 was used for statistical analysis. Data are expressed as the means ± SD. The one-sample Kolmogorov-Smirnov test was used to check for a normal distribution. Student’s t-test was used for between-group comparisons. One-way ANOVA or a Welch test was used for comparisons among groups, and Tukey’s post hoc test was used for multiple comparisons. A P value < 0.05 was considered statistically significant.

## Results

### Myocardial IL-33 and sST protein expression during myocardial I/R

**Fig 1:**
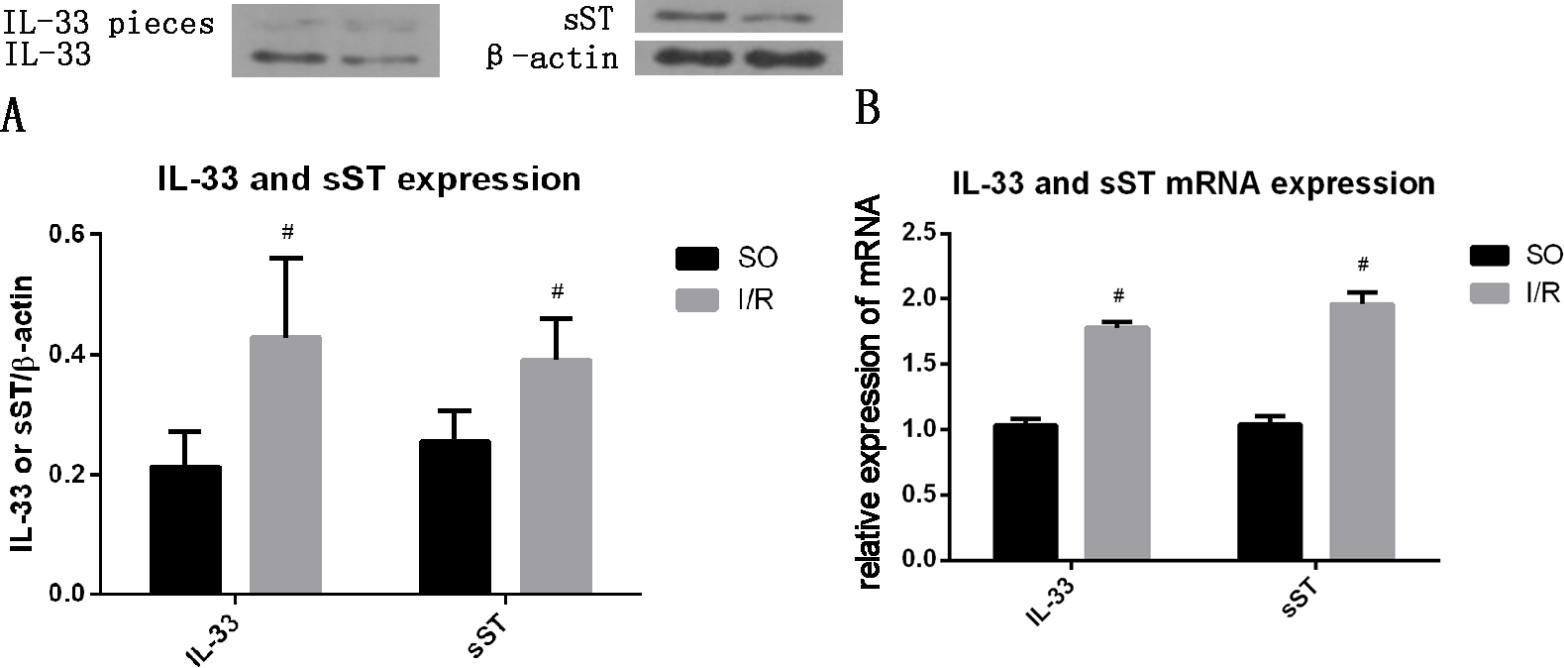
I/R increased the expression of IL-33 and sST in myocardial tissue. (A) Protein level (n = 10), (B) mRNA level (n = 10), #P<0.05.

I/R increased the protein (Fig 1A), and mRNA (Fig 1B) expression (all P<0.05) of IL-33 and sST. A portion of the IL-33 protein was cleaved into two pieces.

### IL-33 reduces myocardial injury after I/R

**Fig 2:**
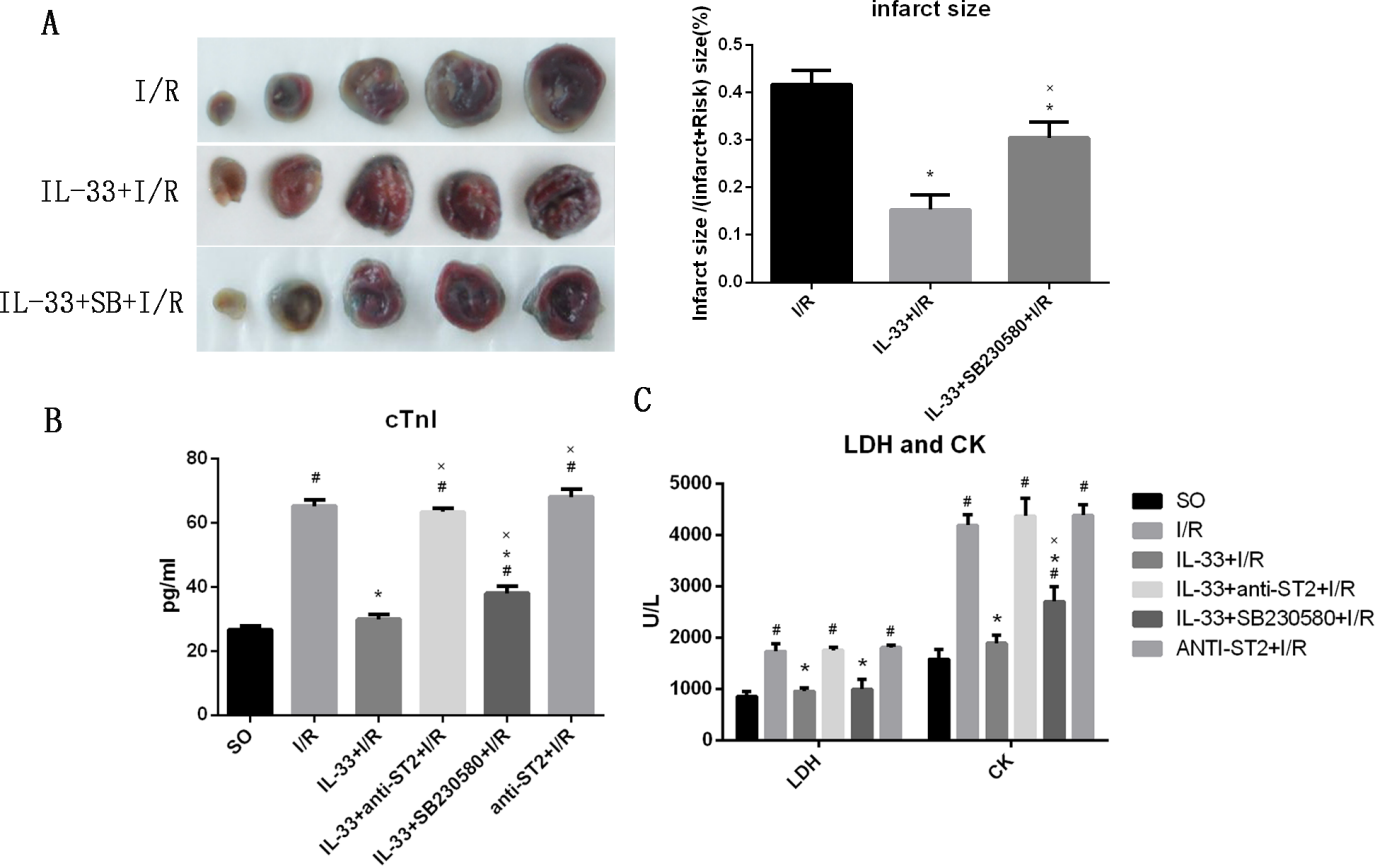
IL-33 reduced myocardial I/R injury, but SB230580 attenuated its effects. (A) The myocardial infarct size (n = 5). (B) The serum level of LDH and CK (n = 6). (C) The expression of cTnI in serum (n = 6). #P<0.05 vs. the SO group, *P<0.05 vs. the I/R group, XP<0.05 vs. the IL-33+I/R group.

To evaluate the effect of IL-33 on myocardial injury, the myocardial infarct size and biomarkers of myocardial damage (cTnI, LDH and CK) were measured, as shown in Fig 2. Compared with the I/R group, the IL-33 group had a significantly smaller infarct (18.2±4.3% vs. 71.8±8.7%, P<0.05). The addition of SB230580 markedly attenuated the effect of IL-33 (44.1±5.1%, P<0.05, Fig 2A). I/R significantly increased the serum cTnI (65.2±1.2 pg/ml), LDH (1738.9±145.9 U/L) and CK (4194.0±206.3 U/L) levels (all p<0.05), and IL-33 attenuated the high expression levels of serum cTnI (29.9±1.4 pg/ml), LDH (958.4±66.5 U/L) and CK (1983.4±154.6 U/L) induced by myocardial I/R (all p<0.05 compared with the I/R group). The addition of SB230580 partially inhibited the effect of IL-33 with regard to increasing the expression level of serum cTnI (38.0±2.6 pg/ml) and CK (2704.3±297.3 U/L) compared with those in the IL-33+I/R group (both P<0.05), but the addition of anti-ST2 completely inhibited the effect of IL-33 (all P>0.05). Compared with the I/R group, the group that received anti-ST2 alone did not show significant effects on the serum cTnI (68.1±2.4 pg/ml), LDH (1809.1±48.1 U/L) and CK (4387.7±200.3 U/L) levels (all P>0.05), but there was an increasing trend (Fig 2B and 2C).

### IL-33 inhibited inflammatory responses

**Fig 3:**
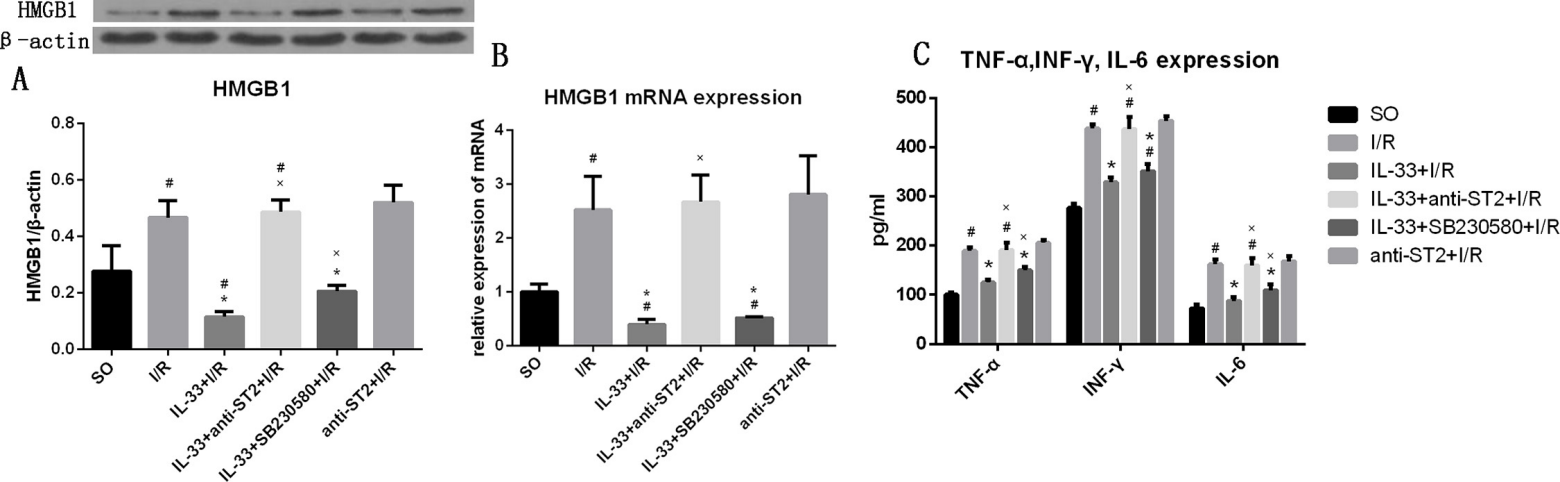
IL-33 reduced the expression of HMGB1 and Th1 inflammatory cytokines (TNF-α, INF-γ and IL-6), but the effects were partially inhibited by SB230580. (A) The expression level of TNF-α, INF-γ and IL-6 in myocardial tissue from infarct and risk areas (n = 6). (B) The level of HMGB1 in myocardial tissue from infarct and risk areas (n = 6). (C) The level of HMGB1 mRNA from infarct and risk areas (n = 6). #P<0.05 vs. the SO group, *P<0.05 vs. the I/R group, ×P<0.05 vs. the IL-33+I/R group.

To evaluate the effect of IL-33 on inflammatory responses in myocardial I/R, the expression levels of HMGB1, TNF-α, INF-γ and IL-6 were measured. Compared with the SO group, the I/R group had higher HMGB1 expression (0.467±0.061 vs. 0.277±0.090, P<0.05). IL-33 attenuated the increased expression of HMGB1 induced by I/R (0.115±0.019, P<0.05), but its effect was completely inhibited by anti-ST2 (0.487±0.042, P<0.05 compared with the IL-33±I/R group; P>0.05 compared with the I/R group). Anti-ST2 inhibited the effect of endogenous IL-33. Compared with that in the I/R group, the expression level of HMGB1 in anti-ST2+I/R group tended to be higher, but the difference did not reach statistical significance (0.521±0.060, P>0.05). SB230580 partially attenuated the effect of IL-33 with regard to decreasing HMGB1 expression (0.206±0.020, P<0.05, Fig 3A). In addition, we found the same trend when the HMGB1 mRNA levels were analyzed (Fig 3B).

Compared with the SO group, the I/R group had significantly higher TNF-α (188.8±8.2 pg/ml), INF-γ (438.2±8.34 pg/ml) and IL-6 (162.1±10.1 pg/ml) expression levels (all p<0.05), and IL-33 attenuated the high expression levels of TNF-α (124.6±5.9 pg/ml), INF-γ (328.5±9.5 pg/ml) and IL-6 (87.5±7.8 pg/ml) induced by myocardial I/R (all p<0.05 compared with the I/R group, and all P>0.05 compared with the SO group). Furthermore, the effect of IL-33 was attenuated by SB230580 (all p<0.05) but was completely blocked by anti-ST2 (all p>0.05). To determine the effect of endogenous IL-33, rats were administered anti-ST2; however, compared with the I/R group, the anti-ST2+I/R group showed no difference in the expression levels of TNF-α (205.4±5.6 pg/ml), INF-γ (453.7±8.85 pg/ml) and IL-6 (167.9±11.4 pg/ml) (all P>0.05, Fig 3C).

### IL-33 suppressed myocardial apoptosis

**Fig 4:**
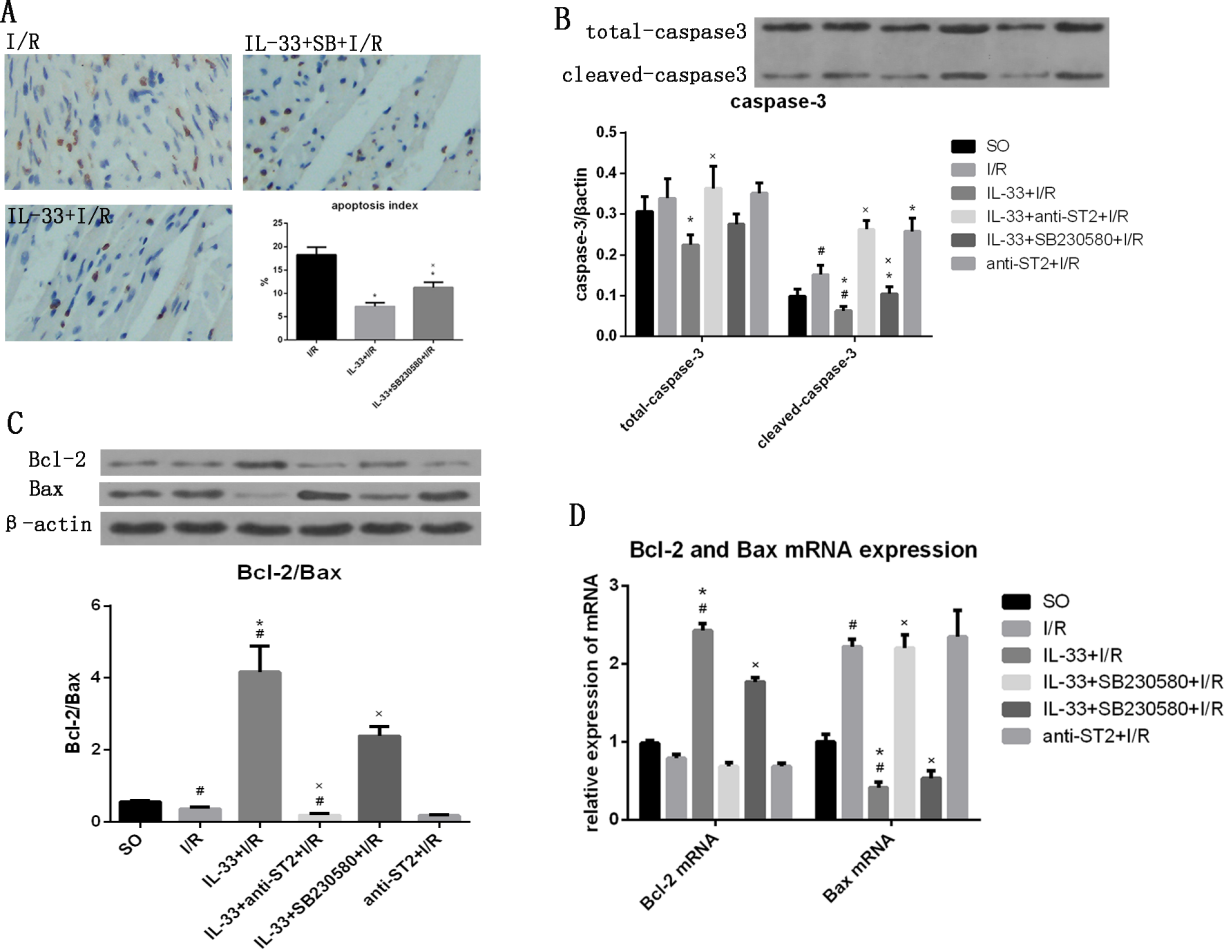
IL-33 suppressed myocardiocyte apoptosis, but the effects were partially inhibited by SB230580. (A) The myocardiocyte apoptosis index of the myocardial tissue from infarct areas (n = 5). (B) The expression levels of total caspase-3 and cleaved caspase-3 in myocardial tissue from infarct and risk areas (n = 6). (C) The expression levels of Bcl-2 and Bax and the Bcl-2/Bax ratio in myocardial tissue from infarct and risk areas (n = 6). (D) Bcl-2 and Bax mRNA expression in myocardial tissue from infarct and risk areas (n = 6). #P<0.05 vs. the SO group, *P<0.05 vs. the I/R group, ×P<0.05 vs. the IL-33+I/R group.

To investigate the effect of IL-33 on myocardial apoptosis in I/R, DNA fragmentation and apoptosis proteins (caspase-3, Bcl-2/Bax) were measured, using TUNEL and western blotting, respectively. IL-33 markedly inhibited I/R-induced myocardiocyte apoptosis (7.2±0.82% vs. 18.3±1.7%, P<0.05), but its effect was attenuated by SB230580 (11.2±1.2%), as shown in Fig 4A. I/R increased the levels of both total caspase-3 (0.339±0.048) and cleaved caspase-3 (0.151±0.023, both p<0.05), and the addition of IL-33 significantly reduced the levels of both total caspase-3 (0.224±0.025) and cleaved caspase-3 (0.063±0.011, both p<0.05). The effect of IL-33 on caspase-3 was attenuated by SB230580 (total caspase-3: 0.276±0.025, cleaved-caspase-3: 0.105±0.017, p<0.05) but was completely inhibited by anti-ST2 (total caspase-3: 0.363± 0.054, cleaved-caspase-3: 0.263±0.022, P<0.05). Anti-ST2 significantly increased the level of cleaved caspase-3 (0.258±0.032, p<0.05) but not total caspase-3 (0.352±0.026, P>0.05, Fig 4B). Compared with the SO group, the I/R group had a lower ratio of Bcl-2 to Bax (0.366±0.054, p<0.05). The ratio was highest in the IL-33+I/R group (4.17±0.72, p<0.05) and lowest in the anti-ST2+I/R group (0.181±0.022, p<0.05). The mRNA expression levels of Bcl-2 presented the same trend, as indicated by quantitative PCR. Bax, which shows the opposite effect of Bcl-2, presented an expression trend opposite to that of Bcl-2 (Fig 4C and 4D).

### IL-33 regulates P38 MAPK

**Fig 5:**
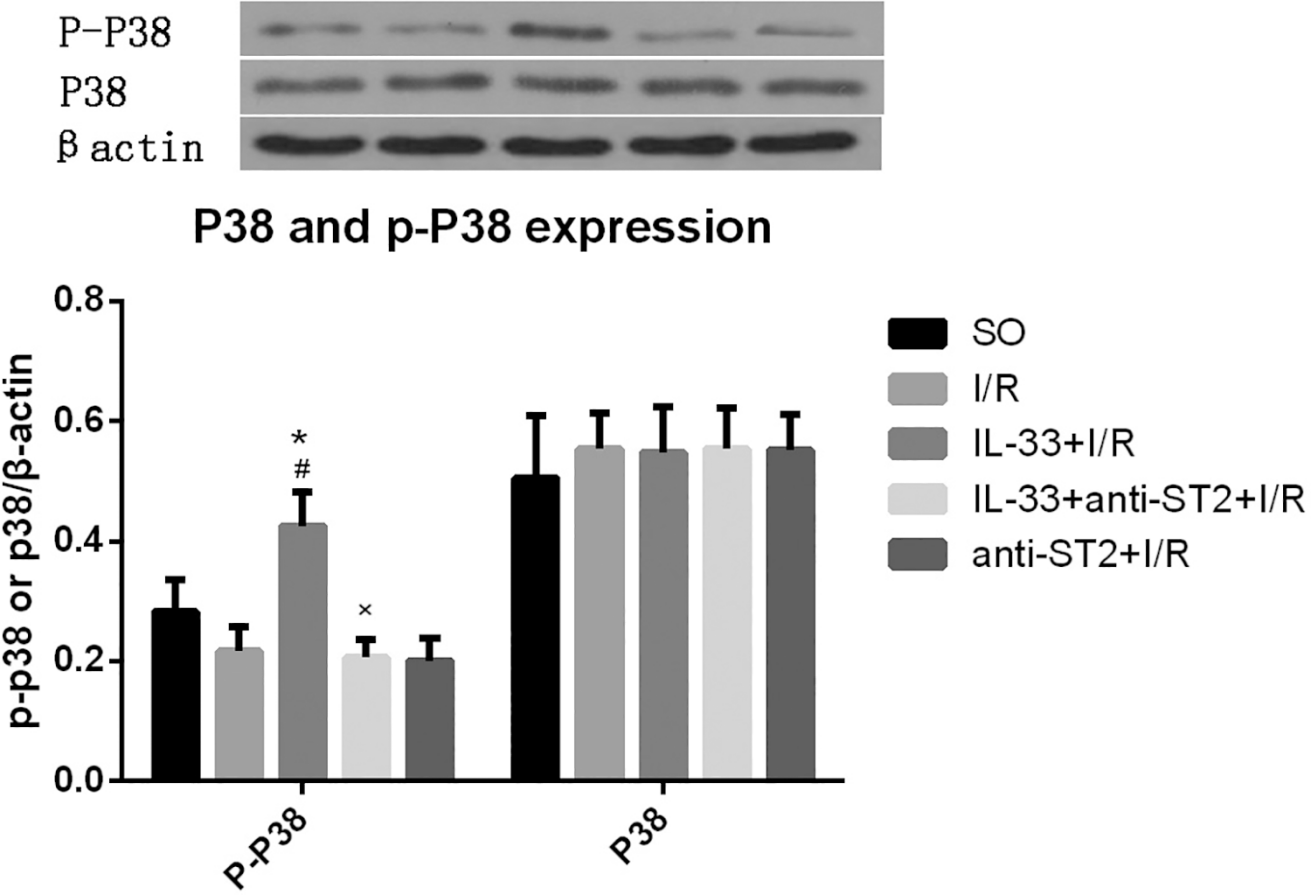
IL-33 activated the P38 MAPK signaling pathway. IL-33 increased the expression level of P-P38 but not the total P38 level in myocardial tissue from infarct and risk areas (n = 6). #P<0.05 vs. the SO group, *P<0.05 vs. the I/R group.

Compared with the I/R group, the IL-33 group had significantly higher expression of P-P38 (P<0.05) but not total P38. Anti-ST2 completely inhibited the effect of IL-33 on p-P38 (Fig 5).

## Discussion

In the present study, we found that I/R significantly increased the levels of IL-33 and sST in myocardial tissue, and a portion of IL-33 was cleaved into two pieces. IL-33, which is the 11^th^ identified IL-1 family member, is expressed in arterial smooth muscle cells, cardiac fibroblasts and the coronary artery endothelium in myocardial tissue [4,16]. During necrosis or apoptosis, IL-33 is released from the nucleus. However, in contrast to the situation in necrotic cells, IL-33 is cut into pieces by cleaved caspase-3 in apoptotic cells. sST, the induced receptor of IL-33, is generally believed to attenuate the effect of IL-33, and its expression is significantly increased after I/R. These findings may explain why anti-ST2 did not significantly affect any indicators other than cleaved caspase-3. In our study, we found that IL-33 inhibited inflammatory responses via suppression of the Th1 cytokine (TNF-α, INF-γ, and IL-6) expression in myocardial I/R. IL-33 is a cytokine with a dual function. It may act as both a transcription regulator in the nucleus and a traditional cytokine when released from nucleus by participating in the inflammation response. Previous studies have demonstrated that IL-33 is an anti-inflammatory cytokine in many cardiovascular diseases and I/R injury, including myocardial I/R injury [6–8,17–20]. Seki et al [6] suggested that IL-33 can induce the Th1 to Th2 shift and inhibit INF-γ expression in a murine myocardial infarction model. Yin et al [18] reached the same conclusion using a murine cardiac allograft model. Our findings are consistent with previous studies. We found that IL-33 suppressed the expression of HMGB1. HMGB1, which is an early pro-inflammatory cytokine, plays a critical role in myocardial I/R [1,13,21]. In addition to directly worsening myocardial I/R injury, HMGB1 can up-regulate other classic pro-inflammatory cytokines, such as TNF-α and IL-6 [1], thus aggravating inflammation and myocardial I/R injury. Our previous study [21] further demonstrated that HMGB1 may worsen myocardial I/R injury in a dose-dependent manner, and inhibiting the expression of HMGB1 by ethyl pyruvate or BNP post-conditioning attenuated inflammatory responses and myocardial I/R injury [13,22]. These results suggested that IL-33 can protect cardiomyocytes from inflammatory responses and I/R injury by inhibiting the release of HMGB1. A previous study suggested that IL-33 could reduce HMGB1 expression by suppressing the nuclear factor-κB (NF-κB) signaling pathway, which is also a downstream signaling pathway of IL-33/ST2 [9,10]. Thus, IL-33 may regulate HMGB1 expression via the NF-κB signaling pathway, but the exact mechanism is still unknown. Apoptosis plays a major role in myocardial I/R injury, and a previous study suggested that inhibition of apoptosis could protect the heart from I/R injury [23]. The present study found that IL-33 significantly increased the ratio of Bcl-2 to Bax and reduced the cleaved-caspase-3 expression and apoptosis index. These findings indicated that IL-33 inhibited myocardial apoptosis during I/R injury, which is consistent with the findings of previous studies [6,7]. Hu et al [21] demonstrated that HMGB1 promoted cell apoptosis in myocardial I/R. Recent studies have further confirmed that HMGB1 regulates cardiomyocyte apoptosis through the HMGB1–TLR4-IL-23-IL-17A axis [24,25]. Thus, we propose that IL-33 inhibits cardiomyocyte apoptosis via regulation of HMGB1 expression. In our study, we further confirmed the conclusions of previous studies, which found that IL-33 is protective against myocardial I/R injury. However, we are the first group to demonstrate that IL-33 protects against myocardial I/R injury by inhibiting the release of HMGB1.

In the present study, we found that the anti-inflammatory and anti-apoptotic effects of IL-33 could be suppressed by inhibiting the P38 MAPK signaling pathway. In addition, IL-33 activated the P38 MAPK signaling pathway. P38 MAPK is a downstream signaling pathway of IL-33/ST2, and it is a classic signaling pathway that participates in various inflammatory responses, including myocardial I/R injury [26]. Yndestad A et al [27] suggested that IL-33 could inhibit the expression of TNF-α and IL-6 in the myocardium via activation of the P38 MAPK signaling pathway. Sakai et al [17] also demonstrated that in liver I/R injury, IL-33 up-regulated the expression of anti-apoptotic proteins, including cyclin D1 and Bcl-2, via activation of the P38 MAPK signaling pathway. Recently, Wang et al [11] and Ha et al [12] demonstrated that the P38 MAPK signaling pathway is involved in HMGB1 release. These results suggest that the P38 MAPK signaling pathway may be involved in HMGB1 release and the protective effect of IL-33 against myocardial I/R injury.

## Conclusions

In conclusion, our study suggests that 1) IL-33 protects against myocardial I/R injury by inhibiting inflammatory responses and attenuating cardiomyocyte apoptosis; 2) IL-33 can suppress the HMGB1 expression in myocardial I/R injury; and 3) P38 MAPK may be involved in the effects of IL-33 with regard to inhibiting HMGB1 expression and may play a role in the protection against myocardial I/R injury.

## Practical Application

The potential applications of IL-33 span a wide range of clinical situations targeting the heart, lung, brain, kidney, liver and skin. Because of its wide expression, IL-33, which is well studied, seems to show an encouraging protective role in various ischemia and reperfusion injury syndromes. Clinical studies [28,29] demonstrated that IL-33 shows an independent association with in-stent restenosis (ISR) after PCI and the adverse outcome in ST-elevation myocardial infarction (STEMI). Our study demonstrated the following: Endogenous IL-33 was insufficient to reverse the local inflammatory response and cell necrosis and apoptosis; thus, it could only act as an alarm. The addition of exogenous IL-33 should reverse the ISR and outcome in STEMI through up-regulation of P38 MAPK and suppression of HMGB1, but more basic and clinical studies are needed. Our study provides new insight into the treatment and prognosis evaluation of coronary heart disease.

## Supporting Information

S1 Table Minimal dataset. There is 5 pages in this excel and they are the basic data of Fig 1 to Fig 5 accordingly.
(XLSX)
